# Uterine Fibroid of the Posterior Wall Successfully Removed

**Published:** 1870-09

**Authors:** A. Reeves Jackson

**Affiliations:** No. 1026 Wabash Ave.; Chicago, Ill.


A MONTHLY RECORD OF

*Medicine, Surgery, and the Collateral Sciences.*

EDITED BY J. ADAMS ALLEN, M.D., LL.D.; AND WALTER HAY, M.D.

VOL. XXVII.—SEPTEMBER, 1870.—No. 9.

### Original Communications.

ARTICLE I.—*Uterine Fibroid of the Posterior Wall Successfully Removed.* By A. REEVES JACKSON, M.D., Chicago, Ill.

Mary Ann E., the subject of the following case, aged twenty-four years, unmarried, applied Sept. 10, 1869. With the exception of some unimportant dyspeptic symptoms, which appeared two years ago, her health had always been good until within the past seven months. In February last she observed that the menstrual discharge, which had commenced at the age of fourteen, and had always been regular and painless, was more profuse and of longer continuance than ever before. With each succeeding period this symptom of excess became more marked, so that latterly the flow continued about two weeks, being abundant throughout, and followed by an interval of equal length of time during which the patient had leucorrhœa. Under this drain she became greatly anemic, and her health rapidly declined—this being more especially observable during the past four or five weeks. Two months ago—early in the month of July—she became conscious of an enlargement of the lower part of the abdomen, and, as about the same time she commenced suffering from symptoms of irritable bladder, she attributed her increased size to disease of that organ. Locomotion had at no time been interfered with; indeed, as far as her failing powers would permit, she had continued to assist in ordinary household duties. The bowels were habitually constipated, and the appetite greatly impaired.

An external examination of the abdomen revealed the presence of a tumor occupying the epigastric region—freely movable from side to side, hard, globular, smooth, and about the size of the gravid uterus at the fifth month. By the touch, combined with external palpation, the cervix uteri was found to be of normal density, somewhat enlarged and low down in the pelvis; the os uteri was circular, well-defined and sufficiently patulous to admit the extreme tip of the finger. The upper part of the pelvic cavity was occupied by a hard, smooth, inelastic body. With some difficulty I introduced a probe to the depth of four inches into the cavity of the uterus, which was then found to move consentaneously with the tumor. The sound passed in front of the latter and was distinctly perceptible through the abdominal and uterine walls. A flexible male catheter was subsequently introduced as far as seven and a half inches. A fibrous tumor of the womb having been diagnosed, its removal, if practicable, was decided upon. Proceedings for this purpose were accordingly commenced Sept. 13th.

A sponge tent was introduced into the cavity of the cervix, and allowed to remain twenty-four hours. On its removal I was enabled to insert the index finger a little beyond the first joint, but could not reach the tumor. A second and larger tent was therefore introduced and permitted to remain twelve hours. This was withdrawn on the morning of the 15th, at which time the cervical canal was found so well dilated that the finger readily penetrated the os internum, from just above which point I could distinctly feel the tumor starting from the posterior wall with which it was intimately blended. A hand applied at the same time to the abdomen, and a finger subsequently inserted into the rectum, enabled me to gain a very clear idea of its size, shape, position, and degree of mobility.

Menstruation having occurred on the evening of the 15th, all treatment of a local character was suspended until Oct. 2nd.

On the morning of that day the cervix was exposed by means of a Sim's speculum, and the anterior lip having been fixed by the insertion of a tenaculum, the lateral walls of the cervix were cut through quite up to their vaginal attachments. The operation was attended by some hæmorrhage, which soon ceased. A medium sized trocar, its point guided by the finger, was then pushed directly into the substance of the tumor, the direction given to the instrument being upwards and forwards. This operation was painless and almost bloodless, only a few drops following the withdrawal of the instrument. The patient was now ordered to take 10 gr. Pulv. Ergotæ every two hours.

Oct. 3rd. 7 P. M. Has had pain during the day. The cervix uteri is quite soft, and is enlarging above the incision. Dr. Isaac Ott is kind enough to see the case with me, and with his assistance I push the trocar into the lower part of the tumor about two inches, giving to the instrument a different direction from that of yesterday. To continue the Ergot.

Oct. 4th. 10 A. M. The Ergot has produced so much nausea that I am obliged to order its discontinuance. The os uteri has not changed since yesterday.

7 P. M. There has been during the day a little discharge of a grayish substance, thin and bloody.

Oct. 5th. This morning I introduce a bistoury carried flatwise against two fingers through the internal os uteri, and make an incision into the capsule of the tumor about two inches long and an inch deep. A considerable gush of blood follows the operation, but it soon ceases.

Oct. 6th. There has been some oozing of blood since the operation of yesterday, accompanied by the peculiar gray discharge previously mentioned. The os and cervix are but little changed, although the lower part of the tumor is evidently softening.

Oct. 7th. Incise the capsule of the tumor again to-day, making a cut about three inches long and an inch in depth, the operation being followed by a copious discharge of blood which ceases in a few minutes.

Oct. 8th. To-day I introduce a small uterine gauge through the os internum, and, engaging it in the incisions previously made, lacerate the substance of the tumor in various directions.

Oct. 9th. The patient has had much pain during the night and this morning, and has no appetite; tongue coated; pulse 104; tenderness in hypogastric region. The lower portion of the tumor is now quite soft, and there is present an almost constant discharge of grayish matter mixed with blood. Conclude to suspend further operative measures for the present, under the impression that the natural powers will now be sufficient to cast off the growth. In the meantime order the adoption of such tonic and dietetic means as will be most likely to sustain the patient through the ordeal. As the discharge is very offensive the utmost attention to cleanliness is enjoined, and the patient advised to syringe the vagina three or four times a day with a solution of permanganate of potash.

Oct. 15th. The general condition of the patient has much improved. Her appetite is better; tongue clean; pulse 90, soft and full. The bloody grayish discharge continues, and has been daily growing more abundant. The os uteri is open to the size of a silver half-dollar, and is filled with a soft decomposing mass.

Oct. 20th. To-day portions of the softened tumor commence passing away. The patient complains of pain, and the discharge is very abundant and very offensive.

Oct. 21st. Succeed to-day in removing a large portion of the tumor by means of the fingers. Shreds and small masses with copious fluid discharge, all highly offensive, are continually passing off. The general health of the patient is failing. She has no appetite; indeed, expresses the greatest loathing for food. Her pulse is now 120, small, quick and sharp; tongue dry and brown. Still she is not despondent, and has the most sanguine hopes of a favorable issue. Order beef-essence and milk-punch, cleanliness, disinfectants, and free ventilation of her apartments.* Deeming it important to hasten the discharge of the remainder of the mass, I order to be given a teaspoonful of fluid extract Ergot

* This patient, in common with many others that I have seen, could not abide the use of *Carbolic Acid*, for the reason—in which I fully coincide—that its odor is vastly more unpleasant and unenduring than most of those which it is *used to mask and neutralize.*

every two hours, with the view of completing its detachment from the uterine walls.

Oct. 22nd. Am summoned at a very early hour this morning to see my patient (six miles distant) whom I find suffering intense pain, the effect, doubtless, of the action of the Ergot. Find on examination a large portion of the tumor protruding through the os uteri, and at once remove it by means of the fingers. Its texture is now so soft, however, that it gives way on the application of the least force, and after making several vain attempts to pass the fingers above it and thus scoop it away, I am reluctantly compelled to defer the removal of the remainder until I can provide myself with more adequate instruments. Discontinue the Ergot, and give a full dose of Morph. Sulph.

4 P. M. She feels easier. Find another portion of the tumor filling the widely dilated os uteri. Remove as much of it as possible by means of the fingers, and then, by means of the placenta forceps, aided by the finger, I succeed in removing the balance of the growth—a mass somewhat larger than a hen's egg.

From this time onward the patient improved steadily. The discharge of small shreds, together with the characteristic grayish sanies, continued more than a week, diminishing in quantity, however, day by day. Her general condition became rapidly better. On the 24th of November—one month subsequent to my last visit—she reported herself at my office entirely well. The womb had nearly regained its normal size, and the last menstrual period had occupied but four days. Her appetite was excellent; the red color was returning to her lips and cheeks, and she had gained twelve pounds in weight.

Remarks. Not many years have elapsed since it was the common belief that uterine tumors, such as the foregoing, were unamenable to surgical treatment. Indeed, even now, when the results of the numerous operations by Atlee, of Philadelphia, Baker Brown and other idol-breakers are before us, many surgeons not otherwise especially conservative are disposed to regard recoveries after such operations rather in the light of escapes from imposed peril than legitimate results of scientific surgery. Pedunculated growths, it is admitted, are quite proper subjects for removal; but those of an interstitial character and those having sessile attachments, although of precisely the same nature, productive of the same symptoms, as effectual in causing hæmorrhage and destroying health, must be let alone—for it almost amounts to that—to do their work of killing, and why? The reasons assigned for this non-interference are threefold. We are told that

1. Fibroid tumors of the uterus are frequently multiple, and although the prominent one may be removed, the reasonable effect of the operation will be to excite activity and rapid growth in the others; hence, the patient will not be permanently benefitted.

2. The operation is a hazardous one, and may in a few hours or a few days deprive a patient of life which might otherwise be protracted for years.

3. Tumors of this class have occasionally been known to disappear after the menapause, or even before, by a spontaneous process of absorption, inflammation or calcification.

With regard to the first of these statements it seems only necessary to say that, granting its entire correctness, the fact admitted affords no good reason why we should abstain from operating in any case where it is otherwise proper to do so. Surely it would be an anomaly in surgical practice to decline removing a portion of necrosed bone for the reason that the disease might subsequently involve another portion of the same bone. And what surgeon would consider himself justified in refusing to cut down upon and tie a bleeding artery, or to operate for stone in the bladder, because in the one case there might be secondary hæmorrhage, and in the other a recurrence of calculous disease? Yet it seems to me that the cases are so nearly parallel that, in order to be consistent, those who advocate and practice operative measures in these latter classes, cannot advise the withholding of them in those in question.

Besides, although it is true that in some instances there are more than one fibrous tumor present in the same womb, yet the rule undoubtedly is that they are solitary; and the cases of recurrence after removal are far too few in number to enable any one to say that such a contingency should be the slightest bar to operation.

That the operation for the removal of interstitial or sessile uterine fibroids, whatever may be the mode adopted, is exceedingly dangerous, I am quite free to admit. But the knowledge of this fact should do no more than admonish us to exhaust other means that have been recommended and used for the purpose of checking the growth, controlling the hæmorrhage and relieving the pain and other local effects of the neoplasm, *provided it is safe to do so*, before resorting to it. It should not in any case be a reason for declining to operate. So long as the tumor is quiescent or of slow growth, and the hæmorrhages resulting from its presence are slight or so far controllable as to enable us to prevent them from producing dangerous exhaustion, just so long is an operation for its removal safely and therefore properly abstained from. But when this is not the case—when repeated hæmorrhages and pain have so exhausted the patient as to jeopard her life,—and when, as is commonly the case where the growths have attained any great size, the assiduous use of proper means have failed to relieve—then I hold that the surgeon is as much bound to operate as he is to perform craniotomy or the Cæsarian section in certain cases of deformed pelvis, having regard wholly to the end in view and not to the possible danger of the means used.

In the case of Miss E. the course here designated was not pursued. There was no delay whatever. When the nature of the disease was ascertained, and its usual course and the probable effect of remedies pointed out to her, she at once elected to have radical measures resorted to, preferring to assume the risks of the operation with the prospect of cure, to the certainty of many months or years of invalidism with the probable necessity before her of a final resort to an operation under more unfavorable conditions. This determination receiving the sanction of her friends the question was settled.

It is generally conceded that the various remedies that have been advised for the purpose of producing absorption of these morbid growths, are at least unreliable, if not indeed useless. Thomas (*Diseases of Women*, p. 421,) says, "Tumors have in certain instances been known to disappear while drugs have been employed, and perhaps they did so in consequence of their use. But no such effect can be looked for with any confidence. Indeed, with our present experience, such a result must be regarded as decidedly exceptional." Scanzoni says, "We do not remember a single case in which, with the means indicated, we have obtained the complete cure of a fibrous body." And so say all those who have given attention to the subject. Each writer, while expressing his want of faith in the efficacy of Iodine, Iodide and Bromide of Potassium, Mercury, Muriate of Ammonia, etc., yet recommends their use. And such advice and such practice are proper enough, I repeat, so long as it is safe to temporize with the patient, because each experimenter usually desires to ascertain for himself the usefulness or uselessness of a remedy. For this reason, and because, also, some observers have thought they found much benefit follow their employment, trial may be given of any of the articles enumerated above for weeks or months together unless urgent symptoms—hæmorrhage more especially—make manifest to us that further delay is dangerous.

A favorite remedy with Dr. Atlee in these cases is the Muriate of Ammonia in doses of 10 gr. three times daily. I can bear testimony to its apparent efficacy in one case in which, by the advice of that gentleman, I used it in conjunction with the external use of Iodine.

The patient, a married lady, aged 45, had never borne children. Had menorrhagia the last three years. During the past year had seldom been free from a dark bloody discharge, sometimes watery and unusually offensive. A well-defined tumor occupied the whole of the right hypochondriac region, extending from the crest of the ilium to an inch and a half above the umbilicus. It was irregularly globular, hard, nodulated, and movable. The sound, passed through a small circular contracted os, entered the uterine cavity to the depth of four and a half inches, and motion then applied to the growth was distinctly transmitted to the instrument, and *vice versa.* Dr. Atlee saw the case in consultation, and diagnosed fibrous tumor of the uterus. The treatment consisted in the administration of Ammon. Murias. gr. x, three times a day, and the daily application externally of the Ungt. Iodini Comp. This treatment was persevered in more than a year, during which time the hæmorrhages ceased entirely, the patient regained her general health, and there was a very sensible diminution in the size of the tumor. Treatment was discontinued in the Spring of 1864, and the patient continued well until within a few months. Quite recently I learned that the tumor had again enlarged and that hæmorrhage had reappeared. Since that time I have made use of the remedy in a number of other cases of the same character, but without being able to perceive that it exerted any influence in controlling any of the symptoms.

Mr. T. Spencer Wells thinks that the use of the Chloride of Calcium has a tendency to produce a condition of atheroma or calcification in the nutrient vessels of these growths, and that thus the size of these latter may possibly be lessened, or at least that their presence may be made innocuous. I have never made use of the remedy, and cannot therefore speak of its merits from personal experience, but coming to us, as it does, with the indorsement of such high authority, it is deserving of consideration and trial.

No. 1026 Wabash Ave.

